# Comparative symptomatology of infection with SARS-CoV-2 variants Omicron (B.1.1.529) and Delta (B.1.617.2) from routine contact tracing data in England

**DOI:** 10.1017/S0950268822001297

**Published:** 2022-08-17

**Authors:** Alice K. E. Ekroth, Piotr Patrzylas, Charlie Turner, Gareth J. Hughes, Charlotte Anderson

**Affiliations:** Health Protection Operations, Field Services, Contact Tracing Data Management and Surveillance, UK Health Security Agency, London, England

**Keywords:** Contact tracing, COVID-19, Delta variant (B.1.617.2), Omicron variant (B.1.1.529), SARS-CoV-2, symptoms

## Abstract

Symptoms are currently used as testing indicators for SARS-CoV-2 in England. In this study, we analysed national contact tracing data for England (NHS Test and Trace) for the period 1 December to 28 December 2021 to explore symptom differences between the variants, Delta and Omicron. We found that at least one of the symptoms currently used as indicators (fever, cough and loss of smell and taste) were reported in 61.5% of Omicron cases and 72.2% in Delta cases, suggesting that these symptoms are less predictive of Omicron infections. Nearly 40% of Omicron infections did not report any of the three key indicative symptoms, reinforcing the importance of the entire spectrum of symptoms for targeted testing. After adjusting for potential confounding factors, fever and cough were more commonly associated with Omicron infections compared to Delta, showing the importance of considering age and vaccination status when assessing symptom profiles. Sore throat was also more commonly reported in Omicron infections, and loss of smell and taste more commonly reported in Delta infections. Our study shows the value of continued monitoring of symptoms associated with SARS-CoV-2, as changes may influence the effectiveness of testing policy and case ascertainment approaches.

## Introduction

The emergence of new SARS-CoV-2 variants has been associated with changes to transmission dynamics and clinical presentation. The emergence of the Alpha variant in September 2020 [1] was associated with a 43–90% increase in observed transmission compared to the original wildtype strain [2]. Similarly, transmission of the Delta variant, detected in April 2021, was found to be 40–60% higher than for Alpha [3]. During December 2021 and January 2022 in England, Omicron emerged, overtaking Delta as the dominant variant [4]. Recognising symptom differences between emerging SARS-CoV-2 variants and other circulating respiratory infections, and how this may vary between groups such as children or vaccinated populations, could help to identify suitable clinical predictors to inform testing policy. In addition, monitoring changes in symptoms will help to better understand the links between genetic diversity, transmission parameters and clinical presentation.

The first reported indicative symptoms of COVID-19 were fever, cough and loss of smell and taste [5, 6]. Since then, a wide range of symptoms have been reported. A UK study explored symptoms of cases from April 2020 to August 2021, from when wild-type, Alpha and then Delta were circulating [7]. The study reported a greater number of Delta cases experiencing fever, headache and sore throat compared to Alpha, but concluded that the changes observed did not support changes to targeted symptom-based testing [7]. Similarly, one London-based survey (up until 11 December 2021) did not identify a clear difference in symptom profile between early Omicron cases and those with Delta infections [8]. They found the most common symptoms associated with a Delta infection were headache for fully vaccinated, sore throat for partially vaccinated and runny nose/sneezing for unvaccinated positive cases, and no clear early symptom difference between this and Omicron [9]. Another UK survey comparing symptomatic PCR-positive and PCR-negative cases found a marked reduction in reporting of loss of smell and taste, and an increase in sore throat. This survey covered PCR-positives from early to late December 2021, when England moved from a dominant Delta to Omicron [10]. However, over the same period sore throat became more commonly reported in symptomatic PCR-negative cases. Taken together with the reduction of previously specific symptoms, this indicates that monitoring of specific symptoms is necessary for effective testing strategies.

Here we report the use of routine data on symptoms collected during contact tracing in England to compare the symptom profile of Omicron and Delta infections to inform testing and response strategies.

## Methods

### Study population and data sources

Case data were obtained from a routine, national contact tracing system (NHS Test and Trace) where cases were defined as people testing positive for SARS-CoV-2 by PCR and referred for contact tracing. Contact tracing in England was a national system, operating at a whole population level where an online form was completed by the case or on the phone by a contact tracer. Symptom data were entered only once and could not be revised following collection. Inputted dates and postcodes were validated at point of entry. Data were obtained from a full extract of the live contact tracing data that were used for analytical purposes. During contact tracing, all cases were asked if they had had or were experiencing any of the following symptoms: fever, cough, shortness of breath, fatigue, altered consciousness, muscle or joint pain, headache, loss of smell or taste, sore throat, runny nose, sneezing, rash, red or irritated eye, loss of appetite, nausea or vomiting, or diarrhoea. A case was defined as symptomatic if at least one symptom was reported at the time of contact tracing.

Variants were defined by genomic sequencing and genotyping of PCR-confirmed cases and detected based on the UKHSA's single and multi-nucleotide polymorphisms variant definitions with targets for VOC-21APR-02 (Delta) and VOC-21NOV-01 (Omicron) genomes [4]. All symptomatic Omicron and Delta cases reporting symptoms between 1 and 28 December 2021 were included, excluding those who did not complete contact tracing or had missing age, sex, geographical region or vaccination status. Vaccination status was determined by linking contact tracing data to the National Immunisation Management System (NIMS) [11] using combinations of NHS number, forename, first initial, surname, date of birth and postcode. Vaccination status was defined by the number of doses received by the time of symptom onset, allowing some delay for protection from the most recent vaccine dose. Categories 1 dose + 21 days, 2 doses + 14 days and 3 doses + 14 days were used, as in UKHSA vaccine surveillance reports [12]. To account for different vaccination programmes for different ages, the exclusion of cases for which no vaccination status was linked was not applied to children under 12 years, as they were unlikely to have been vaccinated. In addition, as the vaccine roll out for those aged 12–18 years started in late September 2021, most cases in this age group were unlikely to have received a third vaccine dose and were therefore considered fully vaccinated if they had received two or three doses.

### Data analysis

The number of symptoms reported by symptomatic cases with each variant was calculated as medians with interquartile ranges (IQR). Differences in reporting of symptoms (days) relative to symptom onset were assessed using a Mann–Whitney *U* test between variants. The number and proportions of cases reporting each symptom were described by variant, age group (0–4, 5–11, 12–18, 19–39, 40–59, ⩾60 years), sex, ethnicity, vaccination status, date of reported symptom onset and geographical region. Differences in the distribution of co-variables and symptoms by variant were assessed using Pearson's *χ*^2^ tests.

Multivariable logistic regression models were used to estimate adjusted associations between reported symptoms and infection with Omicron compared to Delta. Two modelling approaches were adopted: (1) where reported symptom was the outcome and variant the co-variable of interest and (2) where variant was the outcome and each symptom the co-variables of interest. All variables included in the main effects model were selected *a priori*, consistent with other studies which have considered symptom profiles [7, 10, 13]. Associations were adjusted for age group, sex, ethnicity, vaccination status, geographical region and the week in which symptoms began to adjust for potential confounding. Geographical region and the week in which symptoms began were included as proxy indicators for any impact of other circulating respiratory viruses which can cause similar symptoms to SARS-CoV-2 and may have differential activity by region over short time periods. For modelling approach (1) we undertook stratified analysis by age group (0–4, 5–11, 12–18, 19–39, 40–59, ⩾60 years) adjusted for the same co-variables as the main model for all age groups other than 0–4 and 5–11 years where vaccination status was excluded.

The assumption of missingness completely at random for missing data was assessed using Little's Missing Completely at Random (MCAR) test using the function *mcar_test*. Multivariate Imputation by Chained Equation (MICE) was used for imputation of missing values (five replicate datasets). Pooled analysis was used to produce associations and *P* values for imputed data using the *pool* function in the package mice which were compared to the analysis of complete observations only. For modelling approach (2) goodness of fit was assessed using a case classification table with a cut-off of 0.5.

All statistical analysis was carried out in R version 4.1.2 (R Core Team 2021).

## Results

### Descriptive analysis

There were 2 376 049 cases reported to the contact tracing system during the period 1–28 December 2021. Of these, a total of 309 912 were confirmed Omicron and 123 529 confirmed Delta cases. Of these, 224 451 symptomatic Omicron cases and 89 328 symptomatic Delta cases had completed contact tracing. After removal of cases with incomplete data for age (*n* = 7), sex (*n* = 187), geographical region (*n* = 2205) and vaccination status (for those aged 12 or older, *n* = 12 458), the final dataset contained 213 305 (68.8% of total confirmed Omicron cases) Omicron and 85 846 (69.4% of total confirmed Delta cases) Delta cases.

Cases with Delta infection were more likely to be children: 32% of those with Delta were aged under 18 years old, compared to 9.6% of Omicron cases (Table 1). Omicron cases were more likely to have had three vaccine doses compared to Delta cases (21.4–4.6%), and Delta cases (36.9%) were more likely to be unvaccinated than Omicron cases (13.8%). Also, cases with Omicron were more likely to be resident in London (22.5%, compared to 11.0% of Delta cases).

**Table 1.** Descriptive characteristics of Omicron and Delta cases

Characteristic	Category	Delta (%^a^)	Omicron (%^a^)	*χ*^2^ (*P* value)
All cases		85 846 (28.7%)	213 305 (71.3%)	
Age group (years)	0–4	2350 (2.7%)	1660 (0.8%)	313 952 (<0.001)
	5–11	16 691 (19.4%)	6630 (3.1%)	
	12–18	8461 (9.9%)	12 242 (5.7%)	
	19–39	25 434 (29.6%)	107 308 (50.3%)	
	40–59	28 226 (32.9%)	65 244 (30.6%)	
	⩾60	4684 (5.5%)	20 221 (9.5%)	
Sex	Female	45 927 (53.5%)	117 245 (55.0%)	53.0 (<0.001)
	Male	39 919 (46.5%)	96 060 (45.0%)	
Ethnic group	White	72 219 (84.1%)	171 108 (80.2%)	1893.0 (<0.001)
	Asian or Asian British	4775 (5.6%)	15 915 (7.5%)	
	Mixed or multiple ethnic groups	1978 (2.3%)	5629 (2.6%)	
	Black, African, Caribbean or Black British	1274 (1.5%)	8593 (4.0%)	
	Not stated or prefer not to say	4739 (5.5%)	9258 (4.3%)	
	Other ethnic group	861 (1.0%)	2802 (1.3%)	
Vaccination status^b^	1 dose + 21 days	5454 (6.4%)	10 127 (4.7%)	28 223.5 (<0.001)
	2 doses + 14 days	43 887 (51.1%)	127 464 (59.8%)	
	3 doses + 14 days	3957 (4.6%)	45 720 (21.4%)	
	Unlinked	849 (1.0%)	599 (0.3%)	
	Unvaccinated	31 699 (36.9%)	29 395 (13.8%)	
Symptom week^c^	01/12/2021	50 893 (59.3%)	5271 (2.5%)	151 463.0 (<0.001)
	08/12/2021	25 887 (30.2%)	50 151 (23.5%)	
	15/12/2021	7154 (8.3%)	70 906 (33.2%)	
	22/12/2021	1912 (2.2%)	86 977 (40.8%)	
Region	East Midlands	6604 (7.7%)	15 923 (7.5%)	10 866.3 (<0.001)
	East of England	9719 (11.3%)	17 297 (8.1%)	
	London	9479 (11.0%)	47 952 (22.5%)	
	North East	3986 (4.6%)	8013 (3.8%)	
	North West	6908 (8.0%)	26 285 (12.3%)	
	South East	21 668 (25.2%)	48 096 (22.5%)	
	South West	17 557 (20.5%)	22 342 (10.5%)	
	West Midlands	5513 (6.4%)	14 441 (6.8%)	
	Yorkshire and Humber	4412 (5.1%)	12 956 (6.1%)	

a Row percentages.

b Vaccination status was defined by the number of doses received by the time of symptom onset, allowing some delay for protection from the most recent vaccine dose.

c Symptom week encompasses cases reported between Wednesdays listed and the following Tuesdays during the month of December.

The median total number of symptoms reported by cases was 4 for both Omicron and Delta (IQR: 2.0–6.0). There was a significant difference between symptom onset and completing contact tracing between variants (Mann–Whitney *U* test; *W* = 7 434 461 352, *P* < 0.001), where Omicron cases had a median of 3.97 days (IQR: 2.85–5.90 days) and Delta a median of 3.09 days (IQR: 2.03–4.93 days).

For the key symptoms used as SARS-CoV-2 infection predictors (fever, cough and loss of smell and taste), 61.5% of Omicron and 72.1% of Delta cases reported at least one of these symptoms. The crude proportion of cases reporting loss of smell and taste was lower in Omicron (13.4%) than Delta (33.7%). Fever was also reported slightly less often in Omicron (29.2%) than Delta (32.7%). A similar crude proportion of cases reported cough for each variant (Omicron: 44.6%; Delta: 44.0%).

The most common symptom reported was headache, with 56.2% for Omicron and 55.5% for Delta. The next most common symptom for Omicron was sore throat (53%), followed by runny nose (51%). For cases with Delta, the next most common symptoms were runny nose (49%) and cough (44%).

Overall, when comparing crude values, we found that all symptoms (except rash) were significantly different between variants (Table 2).

**Table 2.** Symptom frequency and crude and adjusted association of variant with symptoms

Symptom	Delta	Omicron	*χ*^2^ (*P* value)	OR (95% CI)	*P* value	aOR^a^ (95% CI)	*P* value
Sore throat	29 531 (34.4%)	113 552 (53.2%)	8701.3 (<0.001)	2.17 (2.14–2.21)	<0.001	1.89 (1.84–1.94)	<0.001
Fever	28 084 (32.7%)	62 211 (29.2%)	365.7 (<0.001)	0.85 (0.83–0.86)	<0.001	1.17 (1.14–1.20)	<0.001
Cough	37 741 (44.0%)	95 221 (44.6%)	11.3 (<0.001)	1.03 (1.01–1.04)	<0.001	1.13 (1.11–1.16)	<0.001
Diarrhoea	5334 (6.2%)	15 877 (7.4%)	140.4 (<0.001)	1.21 (1.18–1.25)	<0.001	1.07 (1.02–1.12)	0.010
Muscle ache or joint pain	33 476 (39.0%)	95 076 (44.6%)	776.8 (<0.001)	1.26 (1.24–1.28)	<0.001	1.04 (1.02–1.07)	0.001
Rash	1123 (1.3%)	2729 (1.3%)	0.38 (0.540)	0.98 (0.91–1.05)	0.528	1.03 (0.93–1.15)	0.572
Nausea or vomiting	11 105 (12.9%)	26 402 (12.4%)	17.4 (<0.001)	0.95 (0.93–0.97)	<0.001	1.01 (0.97–1.05)	0.616
Fatigue	34 469 (40.2%)	93 366 (43.8%)	327.5 (<0.001)	1.16 (1.14–1.18)	<0.001	0.98 (0.96–1.01)	0.194
Altered consciousness	3641 (4.2%)	9602 (4.5%)	9.7 (0.002)	1.06 (1.02–1.11)	0.002	0.94 (0.89–0.99)	0.029
Headache	47 667 (55.5%)	119 938 (56.2%)	12.2 (<0.001)	1.03 (1.01–1.05)	<0.001	0.92 (0.90–0.94)	<0.001
Loss of appetite	14 796 (17.2%)	35 288 (16.5%)	20.98 (<0.001)	0.95 (0.93–0.97)	<0.001	0.87 (0.84–0.90)	<0.001
Shortness of breath	11 629 (13.5%)	32 312 (15.1%)	125.2 (<0.001)	1.14 (1.11–1.17)	<0.001	0.86 (0.83–0.89)	<0.001
Runny nose	42 050 (49.0%)	109 180 (51.2%)	118.6 (<0.001)	1.09 (1.07–1.11)	<0.001	0.81 (0.79–0.83)	<0.001
Sneezing	30 700 (35.8%)	85 693 (40.2%)	501.1 (<0.001)	1.21 (1.19–1.23)	<0.001	0.81 (0.79–0.83)	<0.001
Red or irritated eye	7848 (9.1%)	15 829 (7.4%)	248.6 (<0.001)	0.80 (0.77–0.82)	< 0.001	0.70 (0.67–0.73)	< 0.001
Loss of smell or taste	28 891 (33.7%)	28 569 (13.4%)	16 190.9 (<0.001)	0.30 (0.30–0.31)	< 0.001	0.23 (0.22–0.23)	< 0.001

OR, odds ratio; aOR, adjusted odds ratio.

a Associations were adjusted for age group, sex, ethnicity, vaccination status, geographical region and the week in which symptoms began, where the reference group was Delta.

### Multivariable analysis

Little's MCAR test indicated that the assumption of missingness completely at random was not met (*P* value < 0.001). For both modelling approaches, results from analysis using imputed values were practically identical to those from the complete case analysis (results not shown). The only noted difference was a very small reduction in aOR for modelling approach (1) for diarrhoea [1.07 (CI 1.02–1.12) to 1.06 (CI 1.01–1.12)]. Given this finding, results are presented herein using data with complete values only.

Significant differences in the symptom profile between cases infected with the Omicron and Delta variants of SARS-CoV-2 were observed in routine contact tracing data. After adjustment for age group, sex, ethnicity, vaccination status, geographical region and symptom onset date (Table 2), 13 out of 16 symptoms were found to be significantly associated with Omicron or Delta variants. Although statistical significance needs careful interpretation for analysis of a dataset of this size, only three (rash, nausea or vomiting, fatigue) were found to have no observable differences in reporting frequency according to variant.

**Fig. 1.**
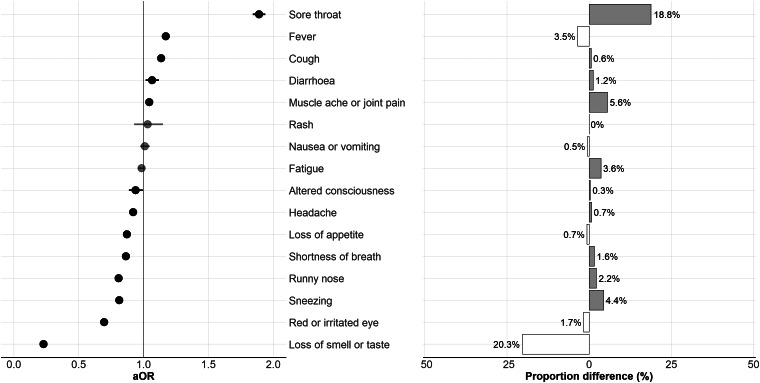
(A) Forest plot of adjusted odds ratios (aOR) for reported symptoms of infection with SARS-CoV-2 Omicron variant compared to Delta variant. Points in solid black indicate a significant result (*P* < 0.05) and points in grey a non-significant result. Error bars indicate 95% confidence intervals. (B) Proportional difference of cases reporting a given symptom, where positive difference (i.e. higher percentage of Omicron cases compared to Delta cases) is shown in grey and negative difference (i.e. higher percentage of Delta cases compared to Omicron cases) in white.

Loss of smell or taste was found to be less common among those with Omicron infections compared to Delta (Fig. 1, aOR 0.23, 95% CI 0.22–0.23, *P* < 0.001). Similarly, runny nose (Fig. 1, aOR 0.81, 95% CI 0.79–0.83, *P* < 0.001), sneezing (Fig. 1, aOR 0.81, 95% CI 0.79–0.83, *P* < 0.001), shortness of breath (Fig. 1, aOR 0.86, 95% CI 0.83–0.89, *P* < 0.001), altered consciousness (Fig. 1, aOR 0.94, 95% CI 0.89–0.99, *P* = 0.032), red or irritated eye (Fig. 1, aOR 0.70, 95% CI 0.67–0.73, *P* < 0.001) and loss of appetite (Fig. 1, aOR 0.87, 95% CI 0.84–0.90, *P* < 0.001) were positively associated with Delta infection. Crude proportions suggested that headache was similarly reported between variants. However, after adjustment, headache was positively associated with Delta infections (Fig. 1, aOR 0.92, 95% CI 0.90–0.94, *P* < 0.001).

We found sore throat (Fig. 1, aOR 1.89, 95% CI 1.84–1.94, *P* < 0.001) was more strongly associated with Omicron infection. Although the crude proportion was lower, after adjustment we found that fever was more commonly reported by Omicron cases compared to those with Delta (Fig. 1, aOR 1.17, 95% CI 1.14–1.20, *P* < 0.001): this had been confounded by age and vaccination status. Crude proportions suggested that cough was similarly reported between variants, however, after adjustment, it was found to be more commonly associated with Omicron infection (Fig. 1, aOR 1.13, 95% CI 1.11–1.16, *P* < 0.001). Finally, muscle ache or joint pain (Fig. 1, aOR 1.04, 95% CI 1.02–1.07, *P* < 0.001) and diarrhoea (Fig. 1, aOR 1.07, 95% CI 1.02–1.12, *P* = 0.010) were slightly more commonly reported by Omicron cases.

The findings from modelling approach (2) are consistent of those from modelling approach (1). Adjusted associations between variant and symptoms and conversely, symptoms with variant, are similar in direction and relative magnitude (Table 3). Evaluation of fit for modelling showed high specificity (0.912) but low sensitivity (0.431), indicating that symptoms alone (and adjustment for important co-variables) are not able to adequately distinguish between infection with Delta and Omicron variants.

**Table 3.** Crude and adjusted associations for symptoms of infection with Omicron variant compared to Delta variant

Symptom	OR (95% CI)	*P* value	aOR (95% CI)	*P* value
Sore throat	2.17 (2.13–2.21)	<0.001	1.87 (1.84–1.91)	<0.001
Fever	0.85 (0.83–0.86)	<0.001	1.09 (1.07–1.11)	<0.001
Cough	1.03 (1.01–1.04)	0.001	1.03 (1.01–1.05)	0.001
Diarrhoea	1.21 (1.18–1.25)	<0.001	1.26 (1.22–1.31)	<0.001
Muscle ache or joint pain	1.26 (1.24–1.28)	<0.001	1.03 (1.01–1.06)	0.002
Rash	0.98 (0.91–1.05)	0.528	1.20 (1.10–1.30)	<0.001
Nausea or vomiting	0.95 (0.93–0.97)	<0.001	1.10 (1.07–1.14)	<0.001
Fatigue	1.16 (1.14–1.18)	<0.001	1.05 (1.03–1.08)	<0.001
Altered consciousness	1.06 (1.02–1.11)	0.002	1.08 (1.04–1.13)	0.001
Headache	1.03 (1.01–1.04)	<0.001	0.84 (0.83–0.86)	<0.001
Loss of appetite	0.95 (0.93–0.97)	<0.001	1.12 (1.09–1.15)	<0.001
Shortness of breath	1.14 (1.11–1.17)	<0.001	1.01 (0.98–1.04)	0.393
Runny nose	1.09 (1.07–1.11)	<0.001	0.80 (0.78–0.82)	<0.001
Sneezing	1.21 (1.19–1.23)	<0.001	0.91 (0.89–0.93)	<0.001
Red or irritated eye	0.80 (0.77–0.82)	<0.001	0.77 (0.74–0.80)	<0.001
Loss of smell or taste	0.30 (0.30–0.31)	<0.001	0.23 (0.23–0.24)	<0.001

OR, odds ratio; aOR, adjusted odds ratio.

### Stratified analysis

**Fig. 2.**
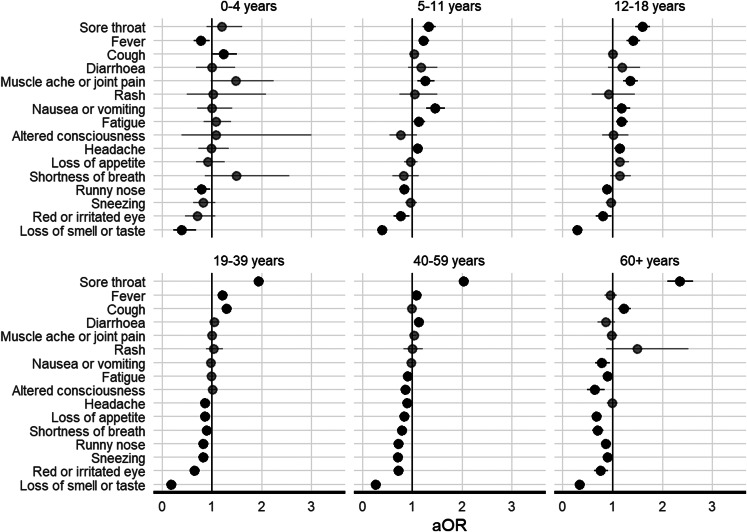
Forest plots of age-stratified analysis showing adjusted odds ratios (aOR) for reported symptoms of infection with SARS-CoV-2 Omicron variant compared to Delta variant. Points in solid black indicate a significant result (*P* < 0.05) and points in grey a non-significant result. Error bars indicate 95% confidence intervals. Vaccination status for ages 0–11 was excluded from the model.

Variations in symptom profile between cases infected with the Omicron and Delta variants were observed in some age groups (Fig. 2). After conducting a stratified analysis by age group, we found that children under 18 years had different symptom profiles compared to adults. However, sore throat was still more commonly associated with Omicron infection and loss of smell and taste with Delta infection across all age groups (Supplementary Table S1).

Variations in reported symptoms were found between some age groups. We found that, whilst fever was more commonly reported in Omicron cases, this was not observed in children under 5 years or adults ⩾60 years (Fig. 2). Also, cough was more commonly associated with Omicron infection in adults (apart from 40–59 years) but not in children under 18 years (Fig. 2). Headache was more common in children over 4 years with Omicron infections and adults with Delta infections (Fig. 2). Whereas, muscle ache or joint pain was reported amongst children aged 5–18 with Omicron, with no evidence of a difference between variants in other age groups (Fig. 2). Shortness of breath, sneezing and loss of appetite were more common in all Delta adult cases (⩾19 years) (Fig. 2). Other symptoms (rash, nausea or vomiting, red or irritated eye, altered consciousness, diarrhoea) showed little variation between age groups.

## Discussion

With the likely continued emergence of SARS-CoV-2 variants, it is important to monitor for potential changes in clinical presentation of COVID-19. Here, we used data from England's national contact tracing system to compare the symptomatology of cases infected with the Omicron and Delta variants. Collectively, we found that key symptom predictors (fever, cough and loss of smell and taste) were more commonly reported in Delta compared to Omicron cases. However, when symptoms were studied individually, we found that fever and cough were more commonly reported by Omicron cases and loss of smell and taste by Delta cases. However, caution should be used whilst interpreting these symptom profiles, given the tendency for highly statistically significant results when analysing large datasets. Understanding comparative symptom profiles, and the variance that may exist between SARS-CoV-2 variants, will help guide the development (and ongoing revision of) screening tools. For example, temperature screening has often been used as a measure to identify cases [14], however, only around 30% of Omicron cases reported fever. Symptom predictors may be limited in informing testing, where a focus on a wider set of symptoms would be far more beneficial.

We found that sore throat was reported significantly more commonly by cases of Omicron compared to those with Delta. This is consistent with another study of PCR-positive cases in England [10]. However, the same study found sore throat was more commonly reported in symptomatic PCR-negative cases during December, suggesting that sore throat may not be a specific predictor of SARS-CoV-2 infection with Omicron. Common symptoms reported for SARS-CoV-2 are also caused by infection with other respiratory viruses that circulate in the UK (influenza A and B, respiratory syncytial virus, adenovirus, parainfluenza, rhinovirus and human metapneumovirus [15]). As we were unable to adjust for the potential impact of co-infections with these viruses, and as our dataset did not include PCR-negative cases, it is not possible to conclude that sore throat is a specific indicator of Omicron infection and further studies are required. An additional limitation to our study is that symptom data were collected at the time of contact tracing only, around 3–4 days post symptom onset, and additional, later-presenting symptoms could not be captured. Symptoms were also self-reported and subject to bias.

Loss of smell and taste was another symptom highlighted in our study. Individuals with Delta infections were significantly more likely to experience this symptom than individuals infected with the Omicron variant. This was also observed in the University of Oxford and Office for National Statistics studies [10], where loss of smell and taste was reported more frequently for wild-type and Delta variant cases compared to Alpha variant cases. Recent studies using animal models have shown the reduced presence of the Omicron variant in lung and nasal tissue in comparison to the original SARS-CoV-2 virus [16–18]. Indeed, when comparing the relative pathogenesis and viral loads of both variants, viral loads were significantly lower in both lung and nasal tissue extracted from mice [16]. Reduced Omicron replication in the nasal tissue may explain why some symptoms, such as loss of smell, are not as pronounced in Omicron infections.

This study describes the changes in reported symptoms associated with the Omicron variant in England. We found sore throat was more common, and loss of smell or taste less common, among people with an Omicron infection compared to a Delta infection. However, all symptoms were found to be reported in cases for both variants, suggesting that testing should remain widespread for many symptoms. Nearly 40% of people with Omicron did not have one of the three key symptoms still used as likely clinical indicators of COVID-19: ensuring professional and public awareness of the variety of SARS-CoV-2 symptoms is needed to ensure appropriate testing and diagnosis. Headache, sore throat and runny nose were the most frequently reported symptoms for Omicron: these non-specific symptoms are likely to be difficult to use to guide testing. As our cohort included only confirmed cases, further analysis including individuals testing negative is needed. Additionally, despite clear findings that indicate distinct symptom profiles for the two variants included in this analysis, our relatively simple modelling approach was not able to adequately discriminate between variants. Future work may be able to develop accurate (and more sophisticated) prediction models that could aid in the surveillance of variants in the absence of high coverage genomics.

## Data Availability

The data that support these studies were collected as part of a public health response, are considered sensitive and not made publicly available. Reasonable requests for access to anonymised data will be considered by the authors on request.

## Supplementary material

For supplementary material accompanying this paper visit https://doi.org/10.1017/S0950268822001297. click here to view supplementary material
