# Supplementary material for: Comparative symptomatology of infection with SARS-CoV-2 variants Omicron (B.1.1.529) and Delta (B.1.617.2) from routine contact tracing data in England
Source: Epidemiol Infect. 2022 Aug 17;150:e162. doi: 10.1017/S0950268822001297 (PMC9509793; doi:10.1017/S0950268822001297)
Supplement: Supplementary file 1 [file S0950268822001297sup001.docx]

**Supplementary Information for**

**Comparative symptomatology of infection with SARS-CoV-2 variants Omicron (B.1.1.529) and Delta (B.1.617.2) from routine contact tracing data in England**

Alice K. E. Ekroth, Piotr Patrzylas, Charlie Turner^,^ Gareth J. Hughes, Charlotte Anderson

**Corresponding author**: [charlotte.anderson@phe.gov.uk](mailto:charlotte.anderson@phe.gov.uk)

This file contains:

**Table S1**

**Supplementary Table 1:** Age stratified adjusted odds ratio (aOR) of Omicron and Delta cases with 95% confidence intervals (CI), and significance of adjusted analysis.

|  | Age: 0-4 | | Age: 5-11 | | Age: 12-18 | | Age: 19-39 | | Age: 40-59 | | Age: 60+ | |
| --- | --- | --- | --- | --- | --- | --- | --- | --- | --- | --- | --- | --- |
| Symptom | **aOR (95% CI)** | **p value** | **aOR (95% CI)** | **p value** | **aOR (95% CI)** | **p value** | **aOR (95% CI)** | **p value** | **aOR (95% CI)** | **p value** | **aOR (95% CI)** | **p value** |
| Sore throat | 1.20 (0.89-1.61) | 0.226 | **1.33 (1.21-1.47)** | **< 0.001** | **1.59 (1.45-1.75)** | **< 0.001** | **1.94 (1.86-2.01)** | **< 0.001** | **2.03 (1.94-2.12)** | **< 0.001** | **2.34 (2.10-2.61)** | **< 0.001** |
| Fever | 0.78 (0.63-0.95) | 0.013 | **1.23 (1.12-1.34)** | **< 0.001** | **1.41 (1.28-1.55)** | **< 0.001** | **1.21 (1.16-1.26)** | **< 0.001** | **1.09 (1.04-1.14)** | **< 0.001** | 0.95 (0.83-1.08) | 0.447 |
| Cough | 1.23 (1.01-1.50) | 0.042 | 1.03 (0.95-1.13) | 0.442 | 1.00 (0.92-1.09) | 0.994 | **1.30 (1.25-1.35)** | **< 0.001** | 0.99 (0.95-1.04) | 0.685 | **1.22 (1.10-1.36)** | **< 0.001** |
| Diarrhoea | 1.00 (0.68-1.46) | 0.997 | 1.17 (0.92-1.50) | 0.198 | 1.18 (0.91-1.54) | 0.212 | 1.04 (0.97-1.12) | 0.231 | **1.13 (1.04-1.22)** | **0.004** | 0.86 (0.70-1.05) | 0.132 |
| Muscle ache or joint pain | 1.48 (0.98-2.24) | 0.065 | **1.26 (1.10-1.44)** | **< 0.001** | **1.35 (1.21-1.50)** | **< 0.001** | 1.00 (0.96-1.04) | 0.990 | 1.03 (0.99-1.08) | 0.175 | 0.98 (0.88-1.09) | 0.714 |
| Rash | 1.02 (0.50-2.09) | 0.955 | 1.05 (0.74-1.50) | 0.787 | 0.91 (0.58-1.44) | 0.700 | 1.04 (0.88-1.23) | 0.644 | 1.00 (0.83-1.21) | 0.972 | 1.49 (0.88-2.52) | 0.142 |
| Nausea or vomiting | 0.99 (0.70-1.40) | 0.971 | **1.45 (1.28-1.65)** | **< 0.001** | **1.17 (1.02-1.35)** | **0.027** | 0.98 (0.93-1.03) | 0.452 | 0.97 (0.91-1.04) | 0.391 | **0.78 (0.65-0.94)** | **0.008** |
| Fatigue | 1.08 (0.84-1.39) | 0.547 | **1.13 (1.02-1.25)** | **0.016** | **1.18 (1.07-1.30)** | **< 0.001** | 0.99 (0.95-1.03) | 0.637 | **0.91 (0.87-0.95)** | **< 0.001** | **0.89 (0.80-0.99)** | **0.037** |
| Altered consciousness | 1.08 (0.39-3.00) | 0.879 | 0.77 (0.54-1.09) | 0.139 | 1.02 (0.79-1.31) | 0.906 | 1.01 (0.94-1.09) | 0.770 | **0.85 (0.77-0.95)** | **0.003** | **0.64 (0.49-0.84)** | **0.001** |
| Headache | 0.99 (0.73-1.34) | 0.933 | **1.11 (1.01-1.21)** | **0.022** | **1.13 (1.04-1.24)** | **0.005** | **0.86 (0.82-0.89)** | **< 0.001** | **0.90 (0.86-0.94)** | **< 0.001** | 0.98 (0.88-1.09) | 0.737 |
| Loss of appetite | 0.92 (0.68-1.25) | 0.603 | 0.96 (0.83-1.11) | 0.583 | 1.14 (0.99-1.32) | 0.072 | **0.86 (0.82-0.90)** | **< 0.001** | **0.84 (0.79-0.89)** | **< 0.001** | **0.67 (0.58-0.77)** | **< 0.001** |
| Shortness of breath | 1.48 (0.86-2.55) | 0.153 | 0.82 (0.60-1.13) | 0.227 | 1.14 (0.96-1.37) | 0.144 | **0.89 (0.85-0.93)** | **< 0.001** | **0.79 (0.74-0.83)** | **< 0.001** | **0.69 (0.59-0.81)** | **< 0.001** |
| Runny nose | **0.79 (0.65-0.97)** | **0.024** | **0.83 (0.76-0.91)** | **< 0.001** | **0.88 (0.80-0.96)** | **0.005** | **0.83 (0.79-0.86)** | **< 0.001** | **0.72 (0.69-0.75)** | **< 0.001** | **0.86 (0.77-0.95)** | **0.004** |
| Sneezing | 0.82 (0.62-1.07) | 0.145 | 0.97 (0.86-1.09) | 0.615 | 0.96 (0.87-1.07) | 0.490 | **0.82 (0.79-0.86)** | **< 0.001** | **0.71 (0.68-0.74)** | **< 0.001** | **0.89 (0.81-0.99)** | **0.039** |
| Red or irritated eye | 0.71 (0.47-1.07) | 0.104 | 0.77 (0.63-0.94) | 0.011 | **0.80 (0.66-0.98)** | **0.028** | **0.65 (0.61-0.69)** | **< 0.001** | **0.72 (0.67-0.77)** | **< 0.001** | **0.75 (0.62-0.91)** | **0.004** |
| Loss of smell or taste | **0.40 (0.23-0.68)** | **< 0.001** | **0.40 (0.34-0.47)** | **< 0.001** | **0.29 (0.25-0.32)** | **< 0.001** | **0.19 (0.18-0.19)** | **< 0.001** | **0.26 (0.25-0.28)** | **< 0.001** | **0.34 (0.29-0.38)** | **< 0.001** |
